# Functional Disability Due to Chronic Low Back Pain in the Geriatric Population of a Tertiary Care Hospital in North India: A Cross-Sectional Study

**DOI:** 10.7759/cureus.59343

**Published:** 2024-04-30

**Authors:** Harshanand Popalwar, Suman Badhal, Nitish Dhiman, Swapnil Sonune, Chinchu K

**Affiliations:** 1 Department of Physical Medicine and Rehabilitation, All India Institute of Medical Sciences, Nagpur, Nagpur, IND; 2 Department of Physical Medicine and Rehabilitation, Vardhman Mahavir Medical College and Safdarjung Hospital, New Delhi, IND; 3 Department of Anesthesiology, Pain Medicine and Critical Care, All India Institute of Medical Sciences, New Delhi, New Delhi, IND

**Keywords:** musculoskeletal illness and disability, geriatric indian population, functional disability, geriatric adults, chronic low back pain

## Abstract

Background

Chronic low back pain (CLBP) is one of the painful and disabling conditions affecting the young as well as the geriatric population. There is a limited body of research to find out the impact of CLBP and functional disability on geriatric adults in the Indian region.

Aim

This study aims to determine the prevalence of functional disability due to CLBP in the geriatric population and to investigate the correlation between functional disability due to CLBP and other sociodemographic factors.

Methodology

A total of 157 geriatric adults were enrolled in the study, fulfilling the inclusion and exclusion criteria. Basic sociodemographic data, along with a clinical-radiological examination, was recorded. The Numeric Pain Rating Scale (NPRS), the Roland and Morris Disability Questionnaire, and the Quebec Back Pain Disability Scale were used as study tools. Summary measures (frequency, mean, median, etc.) are calculated according to the level of measurement of variables. The point prevalence of functional disability due to CLBP in the geriatric population, along with 95% confidence intervals, has been calculated. The prevalence estimates were estimated and calculated with SD variables using a t-test, chi-square test, or Fisher’s exact test under bivariate analysis. The linear/logistic regression analysis was used to control for the effects of covariates. A significance level of 5% was set for all analyses due to the exploratory nature of the study. Statistical significance was considered at p < 0.05.

Results

According to the Roland and Morris Disability Questionnaire, 29% (N = 46) of the study geriatric participants had a severe disability, 45% (N = 70) had a moderate disability, and 26% (N = 41) had a mild disability. According to the Quebec Back Pain Disability Scale, 34% (N = 53) had scored more than 50, and 66% (N = 104) had scored less than 50. Statistically significant correlations have been found between the level of functional disability and intensity of pain (NPRS score), gender, associated illness, current and past occupation, and clinical diagnosis of CLBP (p < 0.05).

Conclusion

The prevalence of functional disability due to CLBP is higher in the geriatric population. It is associated with many influencing sociodemographic factors like gender, occupation, associated musculoskeletal illness, the intensity of low back pain, and clinico-radiological diagnosis. Early identification and timely interventions to reduce functional disability due to CLBP and associated risk factors are the need of the hour. Regular back muscle exercises, ergonomic modifications, and modification of activities of daily life are recommended to prevent functional disability due to CLBP.

## Introduction

According to the World Health Organization, one in six people worldwide will be 60 years old or older by 2030 [1]. By 2050, the world population over the age of 60 will double to 2.1 billion. The National Policy for Older Persons 1999 defines senior citizens as those aged 60 years and older [2]. The size of India’s elderly population (i.e., people over 60 years of age) is rapidly increasing. According to the 2011 census, there are approximately 104 million elderly people (60 years of age and older) in India, including 53 million women and 51 million men [3]. Back pain is a significant medical, social, economic, and public health problem affecting people. It is a condition that has many causes, affects many people, and has many meanings. In the Western world, people are more concerned about back pain and disability. The prevalence of low back pain (LBP) is also high in India; almost 60% of Indians will experience severe back pain at some point in their lives [4]. Functional assessment is an important part of the evaluation and treatment of LBP. Chronic LBP (CLBP) is one of the most common, misunderstood, and disabling conditions in the elderly. Many older adults function well despite having CLBP, and because age-related symptoms are often not associated with pain (e.g., fatigue, sleep, movement problems, etc.), the specificity of CLBP on functional disability is yet unknown [5]. Working adults with CLBP and work disabilities have been extensively studied for work disability and work-related costs being lost. In contrast, there are very few studies on the effects of CLBP and functional impairment in the elderly [6].

## Materials and methods

The study was conducted at the Department of Physical Medicine and Rehabilitation, Safdarjung Hospital, New Delhi, India.

Aim of the study

This study aims to determine the prevalence of functional disability due to CLBP in the geriatric population and to investigate the correlation between functional disability due to CLBP and other clinic-sociodemographic factors.

Rationale for the study

This study shall focus on an exclusive study group of Indian geriatric adults with CLBP and shall study functional disability caused by the same.

Study duration

The present study was carried out at a tertiary care hospital in north India from July 2020 to June 2023.

Sampling size calculation and sampling technique

The sample size was calculated as 160 geriatric adults using open EPI with a confidence level of 95%. This number was decided concerning previous study design [7], where P = expected proportion in population (48%) and d = absolute error/precision (8%). The calculated sample size is N = 150 with a 5% margin; the sample size has been taken as 160. Consecutive patients visiting the outpatient department of physical medicine and rehabilitation with complaints of CLBP were enrolled in the study. The study was started after the institute’s ethics committee approval (approval number IEC/VMMC/SJH/Project/2020-06/08).

Study tool

The following study tools were used for the present study:

Basic Data Collection

The basic data collected in the assessment sheet includes sociodemographic information, clinical examination, the gathering of available medical records, and clinic-radiological diagnosis.

Numeric Pain Rating Scale (NPRS)

This scale was used to measure the intensity of LBP. The NPRS is easy to administer. Patients reported pain severity on a scale of 0 to 10, with 0 suggesting no pain and 10 suggesting the most severe pain. Scores 1-3 indicated mild pain, 4-6 indicated moderate pain, and 7-10 indicated severe pain.

Roland and Morris Disability Questionnaire (Validated Hindi Version)

The Roland and Morris Disability Questionnaire is a standardized, self-administered tool for disability assessment in patients with CLBP. The validated Hindi version is used in the present study. Official permission was obtained to use this validated Hindi version. The translated Hindi version of the questionnaire showed excellent internal consistency (Cronbach α = 0.989) and excellent retest reliability (intraclass correlation coefficient = 0.978) [8]. The study participants were given the Hindi version printouts and asked to self-fill out the questionnaire. The study participants were asked to tick a statement when it applies to them on that specific day and time. The end score is the sum of the ticked boxes, with the ticked boxes as yes and the empty boxes as no. The score ranges from 0 (no disability) to 24 (maximum disability). In general, a higher score indicates greater disability, and a lower score indicates less disability. For the general quantification of the extent of disability in the current study, a score from 0 to 8 suggests mild disability, 9 to 16 indicates moderate disability, and 17 to 24 indicates severe disability [9].

Quebec Back Pain Disability Scale (Validated Hindi Version)

The Quebec Back Pain Disability Scale is a condition-specific questionnaire developed to measure the level of functional disability for patients with LBP. The translated and validated Hindi version of the Quebec Back Pain Disability Scale was used in this study. Official permission was obtained to use this validated Hindi version. The Hindi-validated version showed remarkable internal consistency (Cronbach α = 0.98) [10]. The Hindi version of the Quebec Back Pain Disability Scale has good test-retest reliability, discriminative and convergent validity, and is appropriate for clinical and research use in Hindi-speaking LBP patients. The Quebec Back Pain Disability Scale consists of 20 daily activities. In every activity, there are six answer categories, measured by using a Likert scale from 0 to 5 (0 = no effort, 5 = not able to). If the patient suffers a lot that day, he scores that activity with a 5; if it gives no problems, a 0. The outcome is obtained by summing the scores for the degree of difficulty in performing the 20 daily activities. These outcomes score within the range of 0 and 100. The higher numbers and scores show greater levels of disability. For the general quantification of the extent of disability concerning the current study, a score of 50% or more or less is considered for quantification.

Inclusion criteria

Geriatric patients of both sexes aged 60 years and older and those having LBP and pain lasting for more than 12 weeks.

Exclusion criteria

Acute LBP, back pain with osteoporotic fracture, malignancy, back pain with neuro-deficits, back pain of infective etiology, ankylosing spondylitis, rheumatoid arthritis, and elderly patients with other physical or mental disabilities.

Bias

Selection bias has been minimized by selecting consecutive patients coming to the outpatient Department of Physical Medicine and Rehabilitation with complaints of CLBP.

Measure of data

After the approval of the ethics committee, the researchers collected the data from elderly patients attending the outpatient clinic of the Department of Physical Medicine and Rehabilitation.

Statistical analysis

We used IBM SPSS Statistics for Windows, Version 23.0 (Released 2015; IBM Corp., Armonk, NY, USA) for statistical analysis. Summary measures (frequency, mean, median, etc.) are calculated according to the level of measurement of variables. The point prevalence of functional disability due to CLBP in the geriatric population, along with 95% confidence intervals, has been calculated. The prevalence estimates were estimated and calculated with SD variables using a t-test, chi-square test, or Fisher’s exact test under bivariate analysis. The linear/logistic regression analysis was used to control for the effects of covariates. A significance level of 5% was set for all analyses due to the exploratory nature of the study. Statistical significance was considered at p < 0.05.

## Results

Out of the 160 enrolled study participants, the data of 157 geriatric adults could be retrieved and analyzed with a response rate of 98.1% (male, N = 69; female, N = 88) and a mean age of 64.28 ± 3.26. According to the Roland and Morris Disability Questionnaire, 29% (N = 46) of the study geriatric participants had a severe disability, 45% (N = 70) had a moderate disability, and 26% (N = 41) had a mild disability. The total disability score was (mean ± SD) 12.78 ± 5.42 (Table 1).

**Table 1:** Levels of functional disability and frequency or percentage among geriatric participants according to the Roland and Morris Disability Questionnaire

Levels of functional disability	Frequency, N	Percentage, %
Mild disability	41	26
Moderate disability	70	45
Severe disability	46	29
Total	157	100
Total score of functional disability (mean ± SD)	12.78 ± 5.42	

According to the Quebec Back Pain Disability Scale, 34% (N = 53) had scored more than 50, and 66% (N = 104) had scored less than 50 with a mean score of 45 ± 17.79 (Table 2).

**Table 2:** Levels of functional disability and frequency or percentage among geriatric participants according to the Quebec Back Pain Disability Scale Questionnaire

Quebec Back Pain Disability Scale score	Frequency, N	Percentage, %
<50	104	66
≥50	53	34
Total	157	100
Mean ± SD	45 ± 17.79	

A total of 23% (N = 36) were professional workers earning their livelihood. Moreover, 25% (N = 38) were inactive concerning their previous profession, and 53% (N = 83) were homemakers. From the previous occupation point of view, 20% (N = 32) had desk jobs, and 29% (N = 45) had fieldwork or hard labor jobs (Table 3).

**Table 3:** Demographic characteristics of the elderly, frequency, and correlation between various demographic characteristics of the geriatric population with CLBP and their levels of functional disability CLBP, chronic low back pain; NS, not significant; S, significant

Demographic characteristics	Frequency, N	Percentage, %	Levels of functional disability			p-value (p < 0.05)
			Mild	Moderate	Severe	
Mean age (years)	64.28 ± 3.26		64.43 ± 3.23	64.08 ± 3.03	64.36± 3.02	0.81, NS
Gender						
Male	69	44	26 (63.5%)	36 (51.5%)	7 (15%)	<0.001, S
Female	88	56	15 (36.5%)	34 (48.5%)	39 (85%)	
Occupation (current)						
Working	36	23	13 (32%)	20 (28%)	3 (6.5%)	<0.001, S
Not working	38	24	15 (36%)	18 (26%)	5 (11%)	
Homemaker	83	53	13 (32%)	32 (46%)	38(82.5%)	
Occupation (past)						
Desk job	32	20	11 (27%)	18 (26%)	3 (6.5%)	<0.001, S
Field/hardworking	45	29	17 (41%)	22 (31%)	6 (13%)	
Homemaker	80	51	13 (32%)	30 (43%)	37 (80.5%)	

As per the clinical history and examination, 17% (N = 27) had hypertension, 14% (N = 22) had diabetes type 2, 8% (N = 13) had hypothyroidism, and 6% (N = 9) had COPD. The associated musculoskeletal illness consists of 31.5% (N = 49) osteoarthritis of the knee, and 11.4% (N = 18) had severe osteoporosis. The duration of CLBP symptoms for 36% of participants (N = 56) ranged from 12 months to 24 months, with a mean ± SD NPRS score of 6.45 ± 1.3. From the clinical-radiological diagnosis point of view, 73% (N = 114) had a diagnosis of lumbar spondylosis, 14% (N = 22) had a prolapsed intervertebral disc, and 13% (N = 21) had a chronic back muscle spasm (Table 4).

**Table 4:** Clinical history, associated illness, diagnosis, duration of pain of the geriatric participants, and correlations between levels of functional disability NS, not significant; S, significant

Clinical history	Frequency, N	Percentage, %	Levels of functional disability			
			Mild	Moderate	Severe	p-value <0.05
Systemic illness						
Nil	84	54	22 (54%)	37 (53%)	25 (54%)	0.51, NS
Hypertension	27	17	10 (24.3%)	13 (18.5%)	4 (9%)	
Diabetes mellitus	22	14	5 (12%)	10 (14%)	7 (15%)	
Thyroid	13	8	0	0	0	
Chronic obstructive pulmonary diseases	9	6	3 (7.3%)	6 (8.5%)	4 (9%)	
Others	2	1	1 (2.4%)	4 (6%)	6 (13%)	
Associated illness						
Osteoarthritis of the knee	49	31.5	11 (27%)	20 (28.5%)	18 (39%)	0.01, S
Osteoarthritis of the hip	2	1	0	2 (3%)	0	
Osteoporosis	18	11.4	1 (2.4%)	5 (7%)	12 (26%)	
Osteopenia	1	0.6	0	0	1 (2%)	
Cervical spondylosis	13	8	5 (12%)	8 (11.5%)	0	
Lumbar spondylosis	3	2	1 (2.4%)	1 (1.5%)	1 (2%)	
Spondylolisthesis	3	2	1 (2.4%)	1 (1.5%)	1 (2%)	
Nil	68	43.5	22(54%)	33 (47%)	13 (28%)	
Duration of pain (months)						
<12 months	52	33	17 (41.4%)	20 (28.5%)	15 (32.5%)	0.07, NS
12-24 months	56	36	18 (44%)	26 (37%)	10 (22%)	
25-36 months	28	18	4 (10%)	15 (21.5%)	9 (19.5%)	
>37 months	21	13	2 (5%)	9 (13%)	10 (22%)	
Clinical diagnosis						<0.001, S
Lumbar spondylosis	114	73	28 (68%)	54 (77%)	54 (77%)	
Prolapsed intervertebral disc	22	14	0	12 (17%)	12 (17%)	
Chronic back spasm	21	13	13 (32%)	4 (6%)	4 (6%)	
Total	N = 157	100%				

The present study findings show statistically significant correlations (p ≤ 0.05) between the levels of functional disability and associated illness. In addition to the above, there is a high statistically significant correlation (p < 0.001) between the severe level of functional disability and gender, current occupation, previous occupation, and clinical diagnosis. Also, the statistical correlations between the NPRS score of the elderly with the Roland and Morris Disability Questionnaire and the Quebec Back Pain Disability Scale score are highly significant (p < 0.001) (Table 5).

**Table 5:** Correlations between the NPRS score of the geriatric participants with CLBP and their levels of functional disability (Roland and Morris Disability Questionnaire) and the Quebec Back Pain Disability Scale CLBP, chronic low back pain; HS, highly significant; NPRS, Numeric Pain Rating Scale

NPRS score	CLBP and their levels of functional disability				
	Mild (N = 41)	Moderate (N = 70)		Severe (N = 46)	p-value <0.05
Mean ± SD	5.58 ± 1.33	6.3 ± 1.37		7.45 ± 1.38	<0.001, HS
NPRS score	Quebec Back Pain Disability Scale score				p-value
	<50 (N = 104)		≥50 (N = 53)		<0.001, HS
Mean ± SD	6.07 ± 1.34		7.18 ± 1.37		

There was no statistically significant correlation found between mean age, systemic illness, and duration of pain.

## Discussion

According to the Indian Department of Social Statistics 2016 report, the elderly (over 60 years) accounted for 21% of all disabled people in India. Among the disabled elderly, there are mobility impaired (25%), visually impaired (25%), and hearing impaired (12%). Moreover, 12% have multiple disabilities. These figures are for permanent disabilities, in which the disabilities of movement contribute 25% [11]. The results of this study showed that 29% of the geriatric participants had severe functional disability due to CLBP, with an average disability score of -12.78. This is a third of the studied population. A systematic review was done by de Souza et al. to estimate the prevalence of LBP and determine the functional disability of the elderly in different Portuguese and English populations between January 1985 and October 2018 in relation to the prevalence of LBP in the elderly population [6]. The prevalence of LBP varied from 21% to 60% in most studies. This review showed the high prevalence of LBP in the elderly and the impact of functional disability on factors important for independence. This systematic review supports the findings of the present study on functional impairment induced by CLBP in the elderly. Kirubakaran and Dongre studied chronic musculoskeletal pain in the elderly in rural Tamil Nadu, India and concluded that the most common site of chronic pain was the knee joint (64.5%), followed by mild back pain (21.7%) [12]. In a study by Wettstein et al., pain intensity, disability, and quality of life were examined in 228 older adults with CLBP [13]. They concluded that there is a “paradoxical” effect of age in patients with CLBP and that disability increases with age in patients with CLBP, and quality of life scores are equal or even higher in older patients. This study supports the findings of current research on increasing disability in the elderly. Regarding the different associations between functional disability and sociodemographic data, the severity of functional disability was found to be associated with women and past and present occupations with a field-dedicated work profile. According to Spyropoulos et al., occupational factors such as poor ergonomics, long working hours, and uncomfortable working positions, as well as psychological factors such as work stress, repetitive work, job satisfaction, and anger, can increase the effect [14,15]. Current research results show a strong correlation between functional disability and occupation. Mirzamohammadi et al. studied the assessment of disability in patients with LBP based on the type of lumbar disease [16]. They found that the resulting Oswestry Disability Index was significantly higher in older patients, those with a higher BMI, more work experience, and smokers. A lower Oswestry Disability Index was found in people who exercised regularly. In this study, there is a significant correlation between the clinical-radiological diagnosis of lumbar spondylosis and functional disability, with a p-value of less than 0.001. Shafshak and Elnemr examined a Visual Analogue Scale (VAS) against an NPRS [17]. They concluded that a VAS or NPRS score of 6 could predict severe disability, while a VAS score of more than 4 and an NPRS score of more than 3 could predict moderate disability. Current study results yield a mean NPRS score of 6.3 ± 1.37 for moderate functional disability (N = 70 participants) and 7.45 ± 1.38 for severe functional disability (N = 46 participants). These values are higher than the study mentioned here. There is also a strong correlation between pain due to CLBP and functional impairment (Roland and Morris Disability Questionnaire and Quebec Back Pain Disability Scale), p ≤ 0.001.

Limitations

Functional disability due to other major musculoskeletal conditions like osteoarthritis of the knee, cervical spondylosis, adhesive capsulitis of the shoulder joint, etc., could not be isolated and measured. Psychological disorders like anxiety, high distress levels, and depression could also lead to functional disabilities, which could not be considered in this study.

## Conclusions

The prevalence of functional disability due to CLBP is higher in the geriatric population. It is associated with many influencing sociodemographic factors, like gender, occupation, associated musculoskeletal illness, the intensity of LBP, and clinico-radiological diagnosis. Early identification and timely interventions to reduce functional disability due to CLBP and associated risk factors are the need of the hour. Regular back muscle exercises, ergonomic modifications, and modification of activities of daily life are recommended to prevent functional disability due to CLBP.

## Figures and Tables

**Figure 1:**
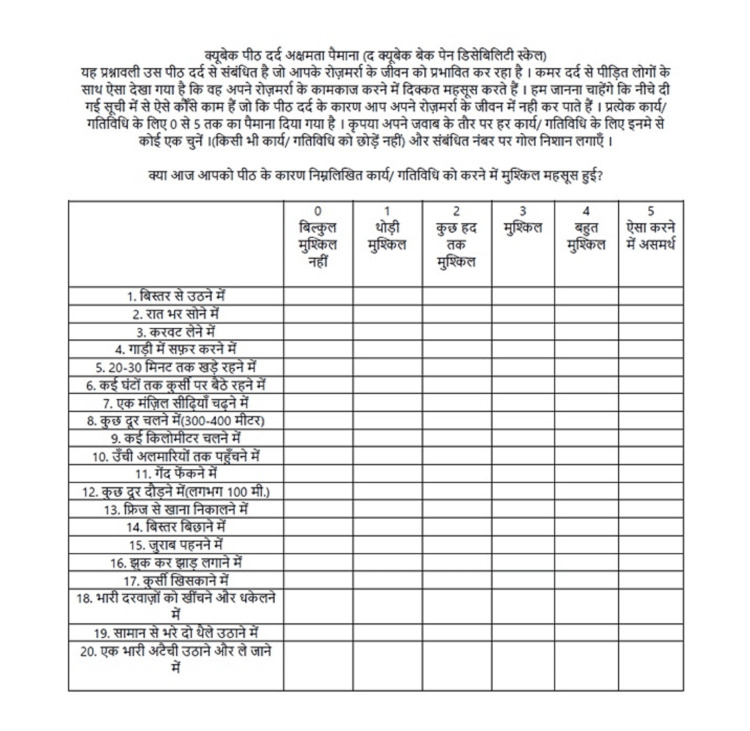
Quebec Back Pain Disability Scale Questionnaire Hindi version

**Figure 2:**
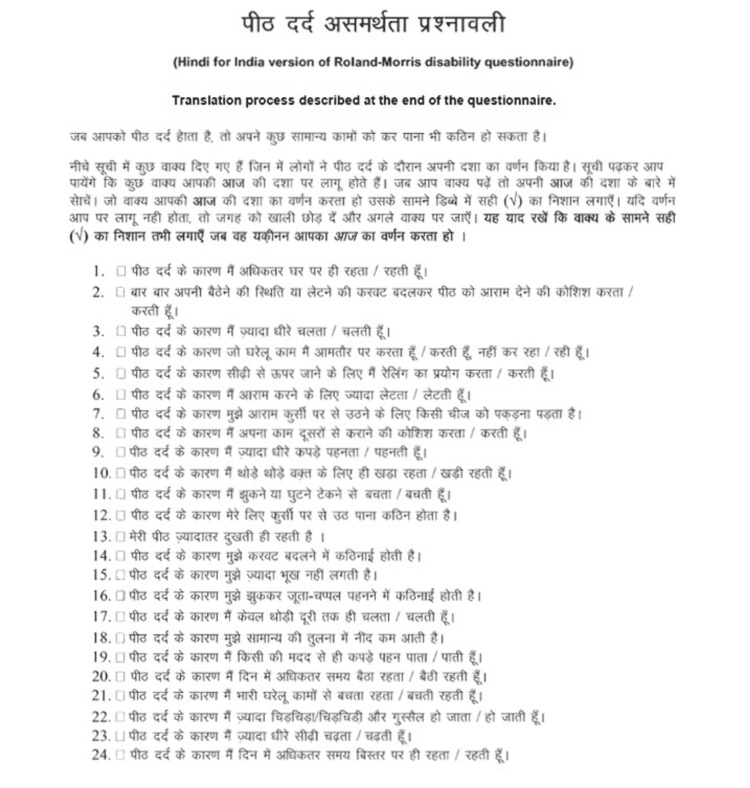
Roland and Morris Disability Questionnaire Hindi version

## Appendices

**Table 6:** Quebec Back Pain Disability Scale

	0, Not difficult at all	1, Minimally difficult	2, Somewhat difficult	3, Fairly difficult	4, Very difficult	5, Unable to do
Get out of bed	0	1	2	3	4	5
Sleep through the night						
Turn over in bed						
Ride in a car						
Stand up for 20-30 minutes						
Sit in a chair for several hours						
Climb one flight of stairs						
Walk a few blocks (300-400 m)						
Walk several kilometers						
Reach up to high shelves						
Throw a ball						
Run one block (about 100 m)						
Take food out of the refrigerator						
Make your bed						
Put on socks (pantyhose)						
Bend over to clean the bathtub						
Move a chair						
Pull or push heavy doors						
Carry two bags of groceries						
Lift and carry a heavy suitcase						

**Table 7:** Roland and Morris Low Back Pain and Disability Questionnaire

I stay at home most of the time because of my back.
I change positions frequently to try to get my back comfortable.
I walk more slowly than usual because of my back. Because of my back,
I am not doing any jobs that I usually do around the house.
Because of my back, I use a handrail to get upstairs.
Because of my back, I lie down to rest more often.
Because of my back, I have to hold on to something to get out of an easy chair.
Because of my back, I try to get other people to do things for me.
I get dressed more slowly than usual because of my back.
I only stand up for short periods of time because of my back.
Because of my back, I try not to bend or kneel down.
I find it difficult to get out of a chair because of my back.
My back is painful almost all the time.
I find it difficult to turn over in bed because of my back.
My appetite is not very good because of my back.
I have trouble putting on my socks (or stockings) because of the pain in my back.
I can only walk short distances because of my back pain.
I sleep less well because of my back.
Because of my back pain, I get dressed with the help of someone else.
I sit down for most of the day because of my back.
I avoid heavy jobs around the house because of my back.
Because of back pain, I am more irritable and bad tempered with people than usual.
Because of my back, I go upstairs more slowly than usual.
I stay in bed most of the time because of my back.
